# Response of airway epithelial cells to double-stranded RNA in an allergic environment

**DOI:** 10.1186/s40247-014-0011-6

**Published:** 2014-09-11

**Authors:** Cristan Herbert, Qing-Xiang Zeng, Ramesh Shanmugasundaram, Linda Garthwaite, Brian G Oliver, Rakesh K Kumar

**Affiliations:** Department of Pathology, School of Medical Sciences, UNSW Australia, Sydney, 2052 Australia; Respiratory Cellular and Molecular Biology, Woolcock Institute of Medical Research, University of Sydney, Sydney, 2037 Australia; Otorhinolaryngology Hospital, The First Affiliated Hospital of Sun Yat-sen University, Guangzhou, 510080 China; School of Medical & Molecular Biosciences, University of Technology Sydney, Sydney, 2007 Australia

**Keywords:** Airway epithelium, Innate interferons, Anti-viral response, Th2 cytokines

## Abstract

**Background:**

Respiratory viral infections are the most common trigger of acute exacerbations in patients with allergic asthma. The anti-viral response of airway epithelial cells (AEC) may be impaired in asthmatics, while cytokines produced by AEC may drive the inflammatory response. We investigated whether AEC cultured in the presence of Th2 cytokines associated with an allergic environment exhibited altered responses to double-stranded RNA, a virus-like stimulus.

**Methods:**

We undertook preliminary studies using the MLE-12 cell line derived from mouse distal respiratory epithelial cells, then confirmed and extended our findings using low-passage human AEC. Cells were cultured in the absence or presence of the Th2 cytokines IL-4 and IL-13 for 48 hours, then stimulated with poly I:C for 4 hours. Expression of relevant anti-viral response and cytokine genes was assessed by quantitative real-time PCR. Secretion of cytokine proteins was assessed by immunoassay.

**Results:**

Following stimulation with poly I:C, MLE-12 cells pre-treated with Th2 cytokines exhibited significantly higher levels of expression of mRNA for the cytokine genes *Cxcl10* and *Cxcl11*, as well as a trend towards increased expression of *Cxcl9* and *Il6.* Expression of anti-viral response genes was mostly unchanged, although *Stat1, Ifit1* and *Ifitm3* were significantly increased in Th2 cytokine pre-treated cells. Human AEC pre-treated with IL-4 and IL-13, then stimulated with poly I:C, similarly exhibited significantly higher expression of *IL8, CXCL9, CXCL10, CXCL11* and *CCL5* genes. In parallel, there was significantly increased secretion of CXCL8 and CCL5, as well as a trend towards increased secretion of CXCL10 and IL-6. Again, expression of anti-viral response genes was not decreased. Rather, there was significantly enhanced expression of mRNA for type III interferons, RNA helicases and other interferon-stimulated genes.

**Conclusion:**

The Th2 cytokine environment appears to promote increased production of pro-inflammatory chemokines by AEC in response to double-stranded RNA, which could help explain the exaggerated inflammatory response to respiratory viral infection in allergic asthmatics. However, any impairment of anti-viral host defences in asthmatics appears unlikely to be a consequence of Th2 cytokine-induced downregulation of the expression of viral response genes by AEC.

**Electronic supplementary material:**

The online version of this article (doi:10.1186/s40247-014-0011-6) contains supplementary material, which is available to authorized users.

## Background

Acute exacerbations of asthma are associated with worsening clinical manifestations requiring a change in treatment strategy [1]. They are the main reason for hospitalisation and the major source of health care costs in asthma [2]. Exacerbations are frequently related to respiratory viral infections, most commonly with human rhinovirus (RV) [3]. Furthermore, asthmatics may develop more severe and longer-lasting RV infections [4],[5].

The airway epithelium is a key player in acute exacerbations of asthma. Not only is it the target of most respiratory viral infections, but it is also an important source of pro-inflammatory cytokines [6]. Several investigators have suggested that one reason for the strong link between exacerbations of asthma and viral infections is that in allergic asthmatics, innate responses to viral infection are impaired. In vitro, there is considerable evidence of decreased production of interferon (IFN)-α2, IFN-β1 and IFN-λ2/3 by airway epithelial cells (AEC) from asthmatics, in response to stimulation with double-stranded RNA (dsRNA) or with RV [7]-[11]. This has been related to impaired toll-like receptor (TLR) and helicase signalling [12]. It has also been suggested that similar impairment is demonstrable in atopic individuals even without asthma [13], although this has not been confirmed.

However, whether the impaired anti-viral cytokine responses translate as increased viral replication in cultures of AEC from allergic asthmatics is much less clear. Although various studies do suggest this [8],[9],[13], others have disagreed [14],[15]. Experimentally, Th2 cytokine pre-treatment of AEC has been reported to increase susceptibility to infection [16],[17] suggested to be related to mucous metaplasia. Again, however, this is controversial, as recent reports have demonstrated either no effect [18] or even that pre-treatment of human AEC with interleukin (IL)-4 and IL-13 was associated with resistance to infection, related to decreased numbers of ciliated cells, with equivalent effect on AEC from asthmatics or non-asthmatics [19].

Another possible reason for the association between viral infections and exacerbations of allergic asthma might be that asthmatic AEC exhibit enhanced expression of pro-inflammatory cytokines in response to viral infection. This has been demonstrated by experimental stimulation with dsRNA, as well by direct infection with viruses including RV [20]-[22]. Furthermore, when stimulated with dsRNA, both asthmatic AEC and normal AEC pre-treated with IL-4 have also been reported to exhibit relatively increased expression of thymic stromal lymphopoietin (TSLP) [10],[23], a cytokine that can induce and amplify Th2 responses.

Overall, however, there remains uncertainty about the nature of the altered responses of AEC to respiratory viral infection in allergic asthmatics, or what might be the mechanism underlying such changes. To further investigate this, we cultured mouse and human AEC in the presence of Th2 cytokines and stimulated them with dsRNA, which is a TLR3 agonist that is also recognised by the RNA helicase *IFIH1* and mimics viral infection [24],[25]. We examined the effect of pre-treatment with Th2 cytokines on the expression of innate and interferon-stimulated anti-viral response genes, as well as of a range of pro-inflammatory cytokines. Our results suggest that a Th2 cytokine environment may promote increased production of pro-inflammatory chemokines by AEC in response to respiratory viral infection, but is unlikely to be responsible for any impairment of anti-viral host defences in asthmatics.

## Methods

### Culture of MLE-12 cells

Preliminary experiments used an SV40-transformed mouse-derived AEC line designated MLE-12 (American Type Culture Collection, Manassas, VA, USA). These cells retain key morphological and functional characteristics of distal airway epithelium [26]. MLE-12 cells were grown in a 50:50 mix of Dulbecco’s Modified Eagle Medium:Ham’s F-12 with 2% heat-inactivated fetal bovine serum and other relevant supplements (L-glutamine, transferrin, sodium selenite, hydrocortisone, β-estradiol, insulin-like growth factor-1 and antibiotics) at 37°C in an atmosphere of 5% CO_2_. Cells were used between passage 2 and 8. To assess responses to poly I:C and the effects of Th2 cytokine pre-treatment, MLE-12 cells were cultured in 25 cm^2^ flasks at 5×10^5^/flask, in media either with or without 20 ng/mL of mouse IL-4 and IL-13 (R&D Systems, Minneapolis, MN, USA) for 48 hours, of which the last 16 hours were in serum-free medium. Cells were then stimulated with 10 μg/mL of poly I:C (Invivogen, San Diego, CA, USA) for 4 hours and total RNA was extracted using TriReagent (Sigma-Aldrich) and stored at −80°C. Five independent experiments were performed.

### Culture of human bronchial epithelial AEC

Approval of all experiments with human lung tissues was provided by the Ethics Review Committee of the South West Sydney Area Health Service, Royal Prince Alfred Hospital and the University of Sydney Human Research Ethics Committee. Bronchial epithelial layers were isolated from 4th-6th order bronchi from lung tissue obtained from 5 patients undergoing lung resection or transplantation (3 with interstitial lung disease, 1 with emphysema, 1 with a neoplasm). Cells were maintained and expanded in Ham’s F-12 with growth supplements as previously described [27]. All experiments were performed with cells at passage 2. AEC were seeded in 6-well plates at a density of 2×10^5^/well in 2 ml BEGM (Lonza, Basel, Switzerland) and incubated at 37°C in an atmosphere of 5% CO_2_. After 16 hours, the medium was changed and cells were cultured either with or without 20 ng/ml of human IL-4 (R&D Systems) and IL-13 (Peprotech, Rocky Hill, NJ) for 48 hours. AEC were then stimulated with 10 μg/ml poly I:C (Sigma-Aldrich) for 4 hours. Culture supernatants were collected and stored at −20°C, while cells were lysed in TriReagent and RNA stored at −80°C.

### Expression of mRNA for cytokines

Quantitative real-time PCR was used to assess the expression of relevant genes, with detection of amplified products using SYBR green (BioLine, Tauton, MA, USA). Primers were designed in-house or sourced from published articles. Reactions were performed using a Roche LightCycler 480 (Roche Diagnostics, Indianapolis, IN, USA), with gene expression normalised to the housekeeping-gene hypoxanthine-guanine phosphoribosyltransferase (HPRT). Each sample was assessed in triplicate.

### Protein immunoassays

For a limited subset of cytokines (CXCL8, CXCL10, CCL5 and IL-6) the concentrations of protein in the supernatants were determined using enzyme-linked immunoassays (R&D Systems) according to the manufacturer’s instructions. Each sample was assessed in duplicate.

### Statistical analysis

Data are presented either as arithmetic means ± s.e.m. (MLE-12 cells) or as before-after plots for individual samples (human AEC). To compare the response of Th2 cytokine pre-treated cells, both unstimulated and following stimulation with poly I:C, changes were assessed by a ratio paired *t*-test, to cater for baseline variability. The software package GraphPad Prism 6.03 (GraphPad Software, San Diego, CA, USA) was used for data analysis and preparation of graphs.

## Results

### MLE-12 cells

Preliminary experiments using these cells revealed that mRNA expression for the chemokine genes *Cxcl10* and *Cxcl11* was significantly increased in cells that had been pre-treated with Th2 cytokines and then stimulated with poly I:C (Table 1). There was also a trend towards increased expression of *Cxcl9* and of the pro-inflammatory cytokine *Il6.* In contrast, levels of expression of the Th2-promoting cytokine *Il33* were significantly decreased in cells that had been pre-treated with Th2 cytokines and then stimulated with poly I:C, while those of *Tslp* were unchanged. Unexpectedly, levels of expression of major anti-viral response genes, including the RNA helicases *Ddx58* (also known as RIG-I), *Ddx60* and *Ifih1* (also known as MDA-5) were mostly unchanged, while the interferon-induced genes *Stat1, Ifit1* and *Ifitm3* were significantly increased in cells pre-treated with Th2 cytokines.

**Table 1:** **Relative expression by MLE-12 cells of mRNA for chemokine, cytokine and interferon-stimulated genes**

	Medium + Poly I:C	Th2 pre-treatment + Poly I:C
*Cxcl1*	2.3 ± 0.3	2.1 ± 0.4
*Cxcl9*	99.0 ± 27.7	178.9 ± 52.7^+^
*Cxcl10*	46.2 ± 29.8	210.5 ± 61.0*
*Cxcl11*	8.6 ± 2.2	61.2 ± 10.8**
*Ccl5*	18.7 ± 2.0	26.8 ± 10.3
*Il6*	1.0 ± 0.4	2.1 ± 0.2^+^
*Il33*	2.3 ± 0.3	1.2 ± 0.2*
*Tslp*	0.5 ± 0.2	0.9 ± 0.4
*Ddx58*	1.2 ± 0.4	1.9 ± 0.7
*Ddx60*	3.5 ± 0.8	5.4 ± 1.2
*Ifih1*	2.8 ± 0.7	3.5 ± 1.7
*Oasl1*	10.4 ± 3.1	9.6 ± 3.8
*Stat1*	3.2 ± 1.9	139.8 ± 30.0**
*Stat2*	1.2 ± 0.5	1.9 ± 0.8
*Ifit1*	4.3 ± 0.8	20.4 ± 7.2*
*Ifitm3*	1.0 ± 0.5	5.6 ± 1.3*

MLE-12 cells stimulated with poly I:C for 4 hours following culture for 48 hours in either medium alone or medium containing IL-4 and IL-13. mRNA expression shown as stimulation ratio (mean ± s.e.m.) relative to cells cultured in medium alone. + 0.05 < *p* < 0.1; *p < 0.05; **p < 0.01 by ratio paired *t*-test, *n* = 5 separate experiments.

### Human AEC

**Figure 1:**
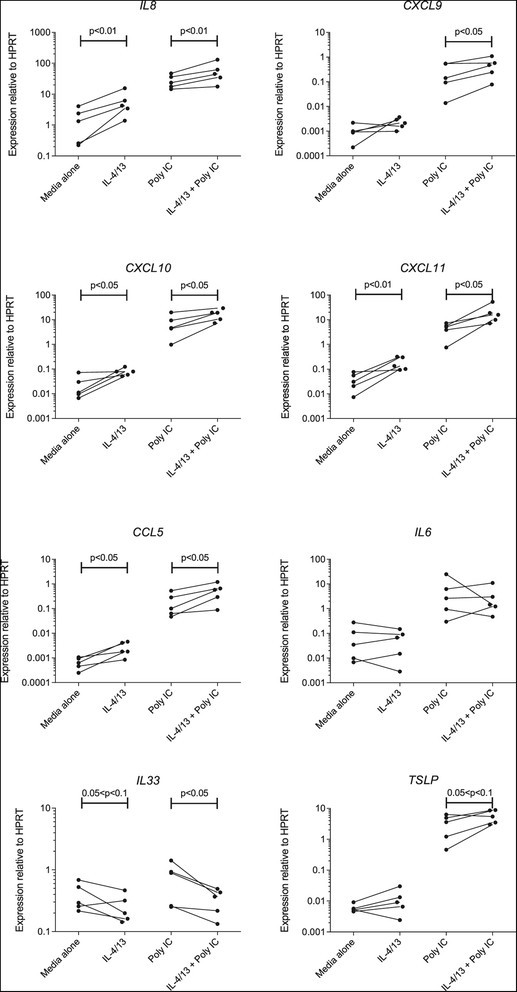
**Before-and-after plots showing effects of prior exposure to Th2 cytokines on the expression of mRNA for chemokine and cytokine genes by human AEC at baseline (left) or following stimulation with poly I:C (right).** Data are mean values for individual patients, showing expression relative to the housekeeping gene HPRT. Note the logarithmic *y*-axis. *p* values for significant differences between cells cultured in media IL-4 and IL-13 were assessed by ratio paired *t*-test.

To confirm and extend these findings, we undertook a comprehensive assessment of the expression of relevant innate interferons, interferon-stimulated anti-viral response genes and pro-inflammatory cytokines by human AEC. As a first step, we showed that cells cultured in the presence of IL-4 and IL-13 exhibited a 2.5-fold increase in expression of mRNA for periostin (expression relative to *HPRT* 0.61 ± 0.14 in media *vs.* 1.56 ± 0.28 in the presence of IL-4/13, *p* < 0.05, unpaired *t-* test), establishing that these cells exhibited a phenotypic change typical of a Th2 environment [28]. Next, we examined the expression of a variety of chemokines and pro-inflammatory cytokines, some of which are known to be interferon-stimulated genes [29]. As shown in Figure 1, baseline levels of expression of the chemokines *IL8*, *CXCL10, CXCL11* and *CCL5* were all significantly higher in cells that had been pre-treated with Th2 cytokines. Furthermore, there was significantly increased expression of *IL8, CXCL9, CXCL10, CXCL11* and *CCL5* in cells that were then stimulated with poly I:C. However, no such increases were observed for *IL6*. Expression of the Th2-promoting cytokine *IL33* was significantly decreased, while there was a trend towards increased expression of *TSLP*.

**Figure 2:**
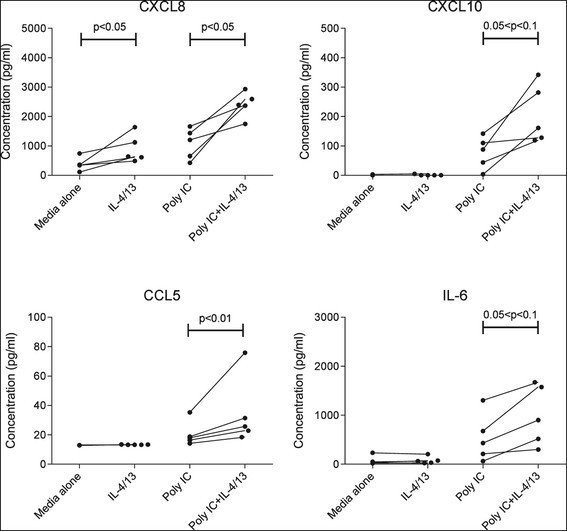
**Before-and-after plots showing effects of prior exposure to Th2 cytokines on the secretion of chemokine and cytokine proteins by human AEC at baseline (left) or following stimulation with poly I:C (right).** Data are mean values for individual patients. *p* values for differences between cells cultured in media with or without IL-4 and IL-13 were assessed by ratio paired *t*-test.

For a limited subset of cytokines, results were confirmed by assessing cytokine protein in culture supernatants, as shown in Figure 2. Interestingly, not only were levels of CXCL8 and CCL5 protein significantly increased, together with a trend towards an increase in levels of CXCL10, but in addition there was also a trend towards elevated levels of IL-6 protein.

**Figure 3:**
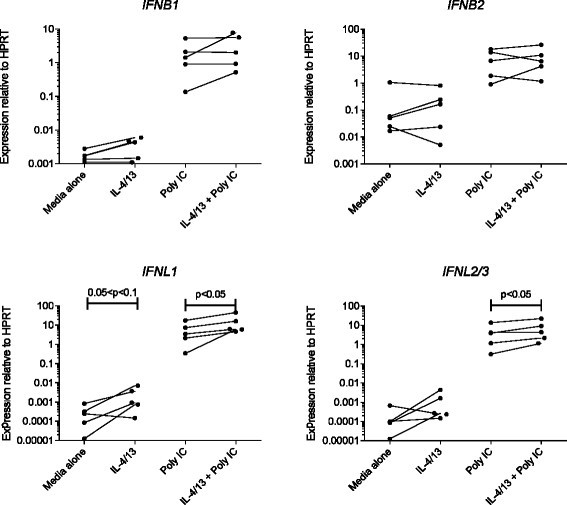
**Before-and-after plots showing effects of prior exposure to Th2 cytokines on the expression of mRNA for type I and type III interferon genes by human AEC at baseline (left) or following stimulation with poly I:C (right).** Data are mean values for individual patients, showing expression relative to the housekeeping gene HPRT. *p* values for significant differences between cells cultured in media with or without IL-4 and IL-13 were assessed by ratio paired *t*-test.

We then examined the expression of innate interferons known to be associated with an anti-viral response. Figure 3 demonstrates that expression of *IFNB1* and *IFNB2* by AEC in response to poly I:C was unchanged in cells that had been pre-treated with Th2 cytokines. However, there was a modest but statistically significant increase in the expression of both *IFNL1* and *IFNL2/3.*

**Figure 4:**
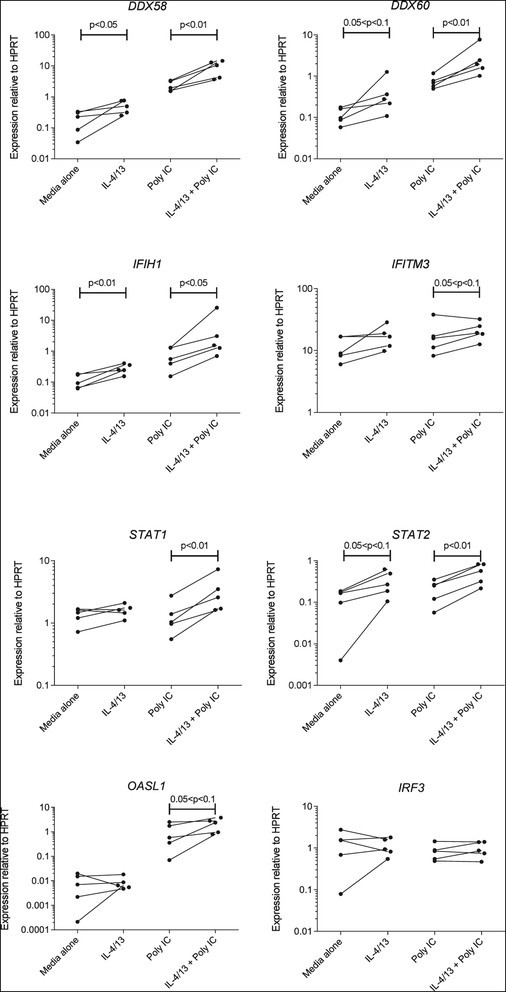
**Before-and-after plots showing effects of prior exposure to Th2 cytokines on the expression of mRNA for anti-viral response genes by human AEC at baseline (left) or following stimulation with poly I:C (right).** Data are mean values for individual patients, showing expression relative to the housekeeping gene HPRT. *p* values for differences between cells cultured in media with or without IL-4 and IL-13 were assessed by ratio paired *t*-test.

Expression of a range of interferon-stimulated anti-viral response genes in cells at baseline or after stimulation with poly I:C is presented in Figure 4. The RNA helicases *DDX58, DDX60* and *IFIH1* were all significantly up-regulated in cells that had been pre-treated with Th2 cytokines and stimulated with poly I:C, while *DDX58* and *IFIH1* was also significantly increased at baseline. In addition, there was a trend towards increased expression of the anti-viral transmembrane protein *IFITM3*. Expression of the transcription factors *STAT1* and *STAT2* was significantly higher, and there was a trend towards increased expression of the transcription factor regulator *OASL1.* However, there was no change in expression of the transcription factor *IRF3*.

## Discussion

In this study, we investigated aspects of the relationship between respiratory viral infections and acute exacerbations of allergic asthma. Using exposure to dsRNA as a surrogate for viral infection, we assessed the effects of prior exposure to Th2 cytokines on the expression by AEC of anti-viral host defence genes including RNA helicases and interferons; signalling pathways that are up-regulated by innate interferons; and various cytokines able to promote an inflammatory response or amplify a Th2 response. In preliminary work using mouse MLE-12 cells, an immortalised line derived from distal AEC, we showed that expression of several chemokines and pro-inflammatory cytokines was significantly up-regulated in cells that had been pre-treated with Th2 cytokines and then stimulated with poly I:C, while expression of major anti-viral response genes was either unchanged or was also significantly increased. This was unexpected and we therefore undertook further work using low-passage human bronchial epithelial cells.

The primary response of AEC to viral infection is the production of interferons, mostly interferon-β1 and the various type III interferons (IFN-λ1/2/3) [30]*.* Because the magnitude of induction of interferons in AEC is relatively low compared to blood leucocytes [30], detection of secreted interferon proteins is difficult, so we assessed expression of these genes by quantitative real-time PCR. We found that in human AEC which had been pre-treated with Th2 cytokines, expression of β interferons was unchanged, while λ interferons exhibited modest but statistically significant up-regulation.

The innate interferons in turn stimulate expression of numerous other genes [29],[31], including not only anti-viral response genes but also chemokines and other pro-inflammatory cytokines, which are secreted at levels that readily permit detection by enzyme immunoassay. Thus we were able to assess the latter in terms of both mRNA expression and protein concentrations in supernatants of AEC in culture. We noted increased expression and secretion of various chemokines, including the neutrophil chemoattractant CXCL8, the T cell chemoattractants CXCL9, CXCL10 and CXCL11, as well as the T cell/eosinophil chemoattractant CCL5. These results were largely similar to the data for MLE-12 cells. Although we observed no change in expression of the *IL6* gene, which is consistent with previously reported data [7], there was some increase in levels of IL-6 protein, possibly indicating secretion of pre-formed cytokine. Interestingly, we observed decreased expression of mRNA for the Th2-promoting cytokine IL-33, again analogous to the finding in MLE-12 cells, while expression of TSLP was increased*.*

Some of the increases in cytokine protein concentrations were not statistically significant, which may have been because culture supernatants were collected at 4 hours after stimulation, a relatively early time point for assessment of secretion of cytokine proteins. Ideally, we would have wished to perform parallel experiments in which cells were collected at 4 hours after stimulation for assessment of mRNA and at 16–24 hours for assessment of protein, but this was not feasible because of the limited availability of human AEC.

With respect to other genes involved in anti-viral defence, we demonstrated up-regulation of the expression of RNA helicases and of the transcription factors STAT1 and STAT2, as well as of other interferon-stimulated genes. However, it was noteworthy that there was no change in the expression of IRF3, even though this transcription factor is believed to be critically involved in the anti-viral response and regulates *IFNB, CXCL9, CXCL10* and *CCL5*[32].

The relationship between respiratory viral infections and asthma is complex, and the underlying mechanisms of cause and effect remain incompletely defined and controversial. For example, there is little doubt that wheezing lower respiratory viral infections in early life are associated with the development of allergic asthma in childhood [33],[34], but it has been suggested that whereas allergic sensitisation increases the risk of wheezing, the converse is not true [35]. Alternatively, some investigators have speculated that development of severe respiratory viral infections is simply an indicator of a genetic predisposition to asthma [36]. Similarly, there is agreement that exacerbations of allergic asthma are most commonly a consequence of viral infections, especially with RV [37]-[39]. However, there is considerable debate about the extent to which an impaired host response might contribute to the development of these infections, or to the severity of infections, or whether the inflammatory response to infection might be significantly different in asthmatics [40].

Our finding of enhanced expression and secretion of a variety of chemokines by AEC pre-treated with Th2 cytokines is consistent with the notion that the allergic environment promotes increased inflammation in response to respiratory viral infection. Our results are concordant with a very recently published study of the response of human AEC to RV, which also demonstrated that cells pre-treated with Th2 cytokines expressed higher levels of the chemokines CXCL8 and CXCL10, independent of any change in viral replication [18]. Increased production of the major neutrophil chemoattractant CXCL8 might help to explain the neutrophilic response to respiratory viral infection observed in the sputum of asthmatics [41],[42]. Increased production of other chemokines might amplify the recruitment of other cell types as well. In this context, it is noteworthy that CXCL10 could be an important pro-inflammatory mediator in asthmatic exacerbations, as it is relatively resistant to suppression by glucocorticosteroids [43].

With respect to epithelial cell-derived Th2-promoting cytokines, the demonstration of a trend towards increased expression of the *TSLP* gene is consistent with earlier evidence that pre-treatment of AEC with IL-4 induces enhanced production of TSLP following exposure to dsRNA [23]. In contrast, decreased expression of IL-33 in AEC pre-treated with Th2 cytokines is somewhat surprising. IL-33 is potentially important in the pathogenesis of exacerbations of asthma [44],[45]. Moreover, it could be released from AEC in response to virus-induced injury (together with other Th2-promoting cytokines such as IL-25 and TSLP) and might thus help to drive airway inflammation in acute exacerbations of allergic asthma [46]. In this setting, because IL-33 behaves in many respects like a damage-associated molecule or alarmin [47], it may be regulated primarily via altered cytokine release, rather than altered expression of mRNA.

Our observation that there was no diminution in the expression of interferons and indeed an increase in the expression of type III interferons contrasts with another in vitro study, which indicated that treatment with IL-13 suppressed production of type III interferons in response to dsRNA by a human AEC line [48]. This issue is pertinent, especially in the context of evidence that asthmatics are more susceptible to develop lower respiratory viral infections [4] and that their infections are of greater severity [49]. Infections in asthmatics have also been reported to persist for longer, although this is controversial and the increase in RV-related illness may instead be a result of re-infection [4],[50]-[53]. Various studies have suggested that impaired production of interferons by AEC from asthmatics, and especially of type III interferons in those with severe asthma, may be an important predisposing factor and may influence the outcome of infection [7]-[10]. Moreover, a deficient type III interferon response has been suggested to play a key role in determining the severity of asthma exacerbations [8]. However, the evidence that interferon production by AEC from asthmatics is impaired is by no means clear-cut [40],[54]. Indeed, it has been suggested that increased levels of type III interferons may play a role in driving virus-induced exacerbations of asthma [55]. Consistent with this, there is no evidence of an increased viral load associated with exacerbations [55],[56].

Our results indicate that any impairment of interferon-mediated defences of airway epithelium in asthmatics is unlikely to be a direct effect of Th2 cytokines on AEC. However, additional factors may operate in vivo. For example, AEC recovered from severe asthmatics have inevitably been exposed to combinations of therapeutic drugs [9] which are recognised to have suppressive effects on host anti-viral and inflammatory responses [57],[58]. Nevertheless, a recent study in an animal model of chronic asthma suggests that long-term allergen challenge may be associated with a decrease in expression of type I and type II interferons, as well as with borderline changes in type III interferons [59]. Intriguingly, these authors also reported decreased production of other pro-inflammatory cytokines, such as IL-1β and IL-12, in response to RV infection.

We recognise the inherent weaknesses of in vitro studies. Furthermore, our experiments utilised undifferentiated immersion cultures of AEC rather than differentiated air-liquid interface cultures. Notwithstanding these limitations, however, we believe that our data shed new light on the complex interplay between respiratory viral infections, the host cytokine response, and acute inflammation of the airways in exacerbations of allergic asthma.

## Conclusions

Collectively, our results suggest that the Th2 cytokine environment which prevails in allergic asthma could promote increased production of pro-inflammatory mediators by AEC in response to respiratory viral infection, but is unlikely to play a role in any impairment of anti-viral host defences in asthmatics.

## Authors’ original submitted files for images

Below are the links to the authors’ original submitted files for images. Authors’ original file for figure 1 Authors’ original file for figure 2 Authors’ original file for figure 3 Authors’ original file for figure 4

## Abbreviations

AEC: Airway epithelial cells
dsRNA: Double-stranded RNA
HPRT: Hypoxanthine-guanine phosphoribosyltransferase
IFN: Interferon
IL: Interleukin
RV: Rhinovirus(es)
TLR: Toll-like receptor
TSLP: Thymic stromal lymphopoietin
