# Supplementary figures and images for: Response of airway epithelial cells to double-stranded RNA in an allergic environment
Source: Transl Respir Med. 2014 Sep 11;2:11. doi: 10.1186/s40247-014-0011-6 (PMC4173067; doi:10.1186/s40247-014-0011-6)

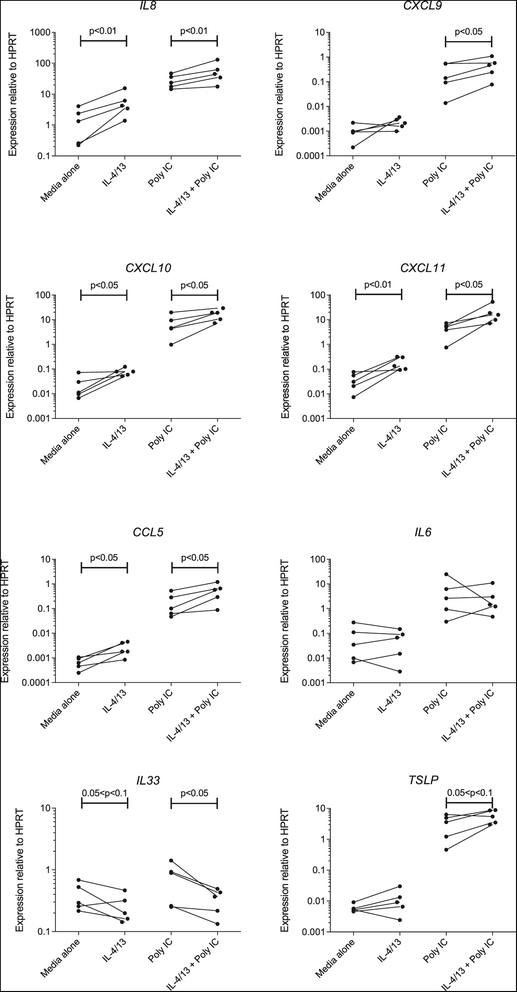

Supplement: Supplementary file 1 — Authors’ original file for figure 1 [file 40247_2014_11_MOESM1_ESM.gif]

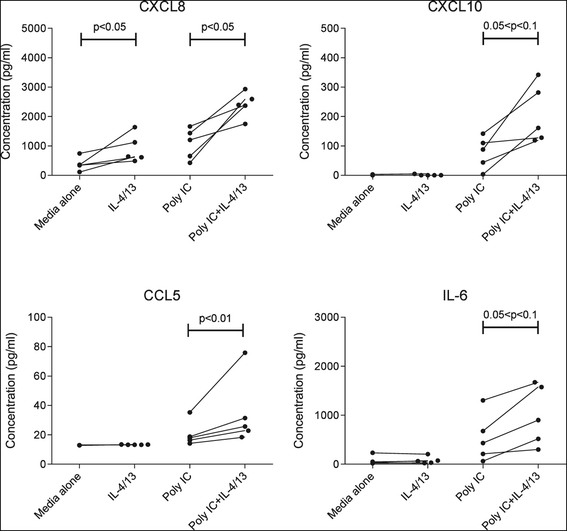

Supplement: Supplementary file 2 — Authors’ original file for figure 2 [file 40247_2014_11_MOESM2_ESM.gif]

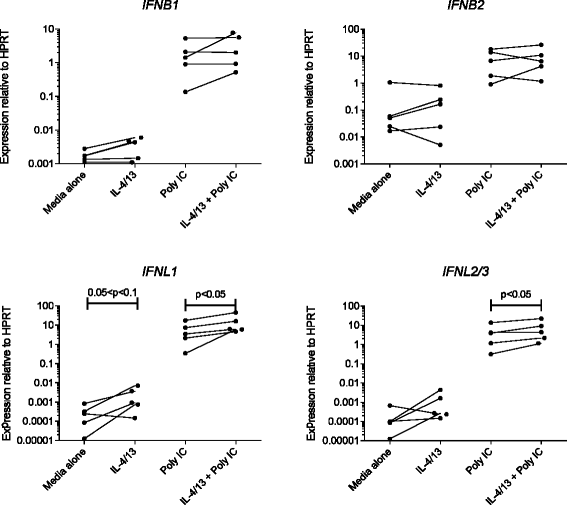

Supplement: Supplementary file 3 — Authors’ original file for figure 3 [file 40247_2014_11_MOESM3_ESM.gif]

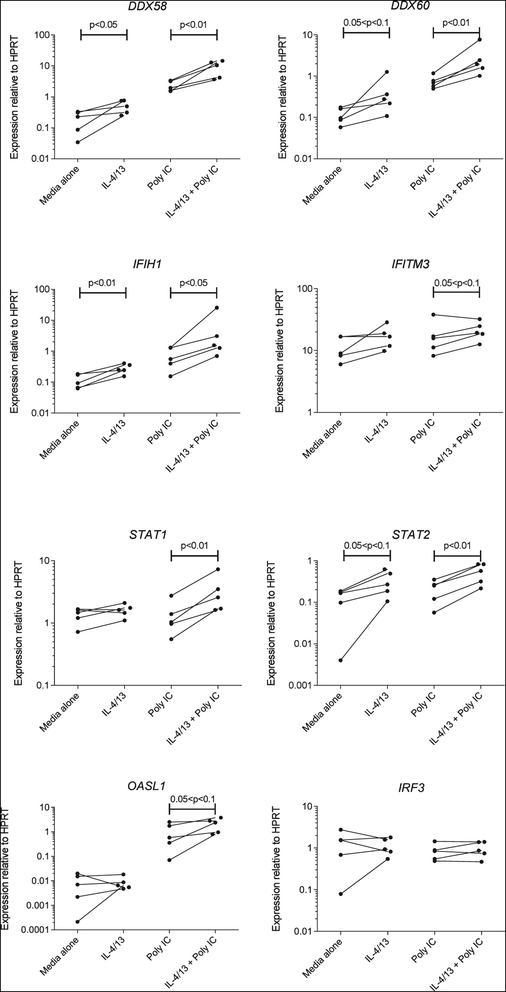

Supplement: Supplementary file 4 — Authors’ original file for figure 4 [file 40247_2014_11_MOESM4_ESM.gif]
